# The Story of the Insane from Year to Year

**Published:** 1896-12-26

**Authors:** 


### THE STORY OF THE INSANE FROM YEAR TO YEAR.

### Ninth Series.

### NORFOLK COUNTY ASYLUM.

The numbers fell from 784 to 774. There were 143 admissions, 32 readmissions, 81 recoveries, 9 discharged relieved, and 80 deaths. The percentage of recoveries on admissions was 53·64, and that of the deaths was 10·28. Brain diseases are responsible for 26 of the deaths, consumption for 11, pneumonia for 8, enteric fever for 1, and enteritis for 1. Of the year's admissions, 37 were driven insane by moral causes such as domestic trouble, religion, love; 15 by drink, and 62 by hereditary influence. The asylum was again visited by colitis and enteric fever, there being two cases of the former and one of the latter. That neither of the diseases spread is fairly indicative that there is not much wrong with the sanitary arrangements. Dr. Thomson was fortunate in having a year pass which was free from serious accident, or suicide, or coroner's inquest. A beginning has been made in the training of nurses and attendants, and several are being prepared for the examination of the Medico-Psychological Association; but Dr. Thomson will probably find that he will have to go far beyond this examination and have to continue the classes. The committee record "their satisfaction with the efficient manner in which Dr. Thomson has managed the establishment." The average weekly cost per head was 9s. 4½d.

THE SOMERSET COUNTY ASYLUM.

A decrease from 891 to 885 has to be noted. There were 181 admissions, 60 readmissions, 90 recoveries, 11 discharged relieved, and 83 deaths. The recoveries stand at 40·54 per cent., calculated on the admissions, and the deaths at 9·39 per cent. on the average number resident. Twenty-eight of the deaths were caused by cerebral and spinal diseases, 15 y consumption, 8 by pneumonia, 3 by bronchitis, and 1 by enteritis. Of the admissions, 26 owed their insanity to adverse circumstances, religion, love, &c., 8 to intemperance in drink, and 28 to hereditary influence. The asylum is full, and 182 patients are boarded out in other asylums at a great additional cost to the ratepayers, and this will go on until the opening of the second asylum, and even then, it is to be feared, the relief will be merely temporary unless either the new asylum or the old one be enlarged almost immediately. Dr. Wade strongly approves of accommodating private patients in public asylums, and it would seem that there is a building at the Somerset Asylum which could readily be transformed into a suitable place for paying patients. Such provision would be a great boon to Somerset, a considerable source of profit to the asylum, and a fruitful cause of worry to Dr. Wade. The visitors say that "Dr. Wade has continued to exercise the greatest care and attention in the general superintendence of the asylum." The weekly cost of maintenance is 8s. 7¾d. per head.

NORTHUMBERLAND COUNTY ASYLUM AT MORPETH.

In this asylum there was a slight fall in the numbers during 1895, there being 599 at the beginning of the year and 583 at the close. There were 136 admissions, 21 readmissions, 69 recoveries, 5 relieved, and 58 deaths. The recoveries stand at 43·8 per cent., and the deaths at 9·8 per cent., the former being, as usual, calculated on the admissions, and the latter on the average number resident. Drink accounts for 26 of the admissions, hereditary tendency for 28, and moral causes for 4. Of the deaths, 24 were caused by cerebral and spinal diseases, and 16 by consumption, the latter being unusually high; but it is hoped that the new warming and ventilating arrangements will reduce the death-rate from that cause. The asylum is again full or nearly so, and 10 male patients are boarded out in the Menston Asylum and 19 at the East Riding Asylum. The chaplain resigned on account of permanent illness, and was granted a pension of £87 10s. per annum. The bricklayer also resigned, and was granted a pension of £40 per annum. The visitors state that Dr. McDowall "continues to carry out his very onerous and responsible duties with every care and attention." The cost per head per week is 9s. 11¼d.

